# Organotins in Neuronal Damage, Brain Function, and Behavior: A Short Review

**DOI:** 10.3389/fendo.2017.00366

**Published:** 2018-01-08

**Authors:** Igor Ferraz da Silva, Leandro Ceotto Freitas-Lima, Jones Bernardes Graceli, Lívia Carla de Melo Rodrigues

**Affiliations:** ^1^Laboratory of Neurotoxicology and Psychopharmacology, Department of Physiological Sciences, Federal University of Espirito Santo, Vitória, Brazil; ^2^Laboratory of Endocrinology and Cellular Toxicology, Department of Morphology, Federal University of Espirito Santo, Vitória, Brazil

**Keywords:** behavioral impairments, brain function, environmental contaminant, endocrine disruptor, neurotoxicity, neurodegeneration, neuroinflammation, oxidative stress

## Abstract

The consequences of exposure to environmental contaminants have shown significant effects on brain function and behavior in different experimental models. The endocrine-disrupting chemicals (EDC) present various classes of pollutants with potential neurotoxic actions, such as organotins (OTs). OTs have received special attention due to their toxic effects on the central nervous system, leading to abnormal mammalian neuroendocrine axis function. OTs are organometallic pollutants with a tin atom bound to one or more carbon atoms. OT exposure may occur through the food chain and/or contaminated water, since they have multiple applications in industry and agriculture. In addition, OTs have been used with few legal restrictions in the last decades, despite being highly toxic. In addition to their action as EDC, OTs can also cross the blood–brain barrier and show relevant neurotoxic effects, as observed in several animal model studies specifically involving the development of neurodegenerative processes, neuroinflammation, and oxidative stress. Thus, the aim of this short review is to summarize the toxic effects of the most common OT compounds, such as trimethyltin, tributyltin, triethyltin, and triphenyltin, on the brain with a focus on neuronal damage as a result of oxidative stress and neuroinflammation. We also aim to present evidence for the disruption of behavioral functions, neurotransmitters, and neuroendocrine pathways caused by OTs.

## Introduction

In recent neurotoxicology studies, there is a growing interest in chemical pollutants with endocrine disruptor properties (1). Endocrine-disrupting chemicals (EDCs) are compounds capable of altering and modulating the normal functioning of the endocrine system, either increasing or blocking the synthesis, release, and action of a natural hormone, or acting like a xenohormone and mimicking the physiological effects of a particular endogenous hormone (1, 2). EDCs with neurological and behavioral effects include bisphenol A, phthalates, pesticides, and organometallic compounds, such as methylmercury and organotins (OTs) (3–5). OTs are organometallic compounds with one or more bonds between a carbon atom and a tin atom. They interfere with the metabolism of the gonadal and metabolic hormones (6, 7) and present cytotoxic and genotoxic effects, notoriously trespassing the blood–brain barrier and presenting neurotoxic effects that lead to nervous system abnormalities (8–11). The outspread use of OTs in the agriculture and industry led to environmental and occupational incidents, as well as their banishment in several countries (12–15). This mini-review aims to summarize the toxicity of the main OTs in the brain and briefly presents what is known about their impact on behavior and on the central nervous system function of experimental animal models and humans.

## Trimethyltin (TMT) Neurotoxicity

Trimethyltin is one of the most commonly used OTs in industry and agriculture, known for its role as a fungicide and plastic stabilizer (16). The symptoms of TMT intoxication in humans have been documented after the report of two cases, as described by Fortemps et al. (17). TMT exposure could be associated with neurological disorders, such as headaches, vigilance loss, disorientation, memory deficits, and tonic–clonic seizures. TMT also leads to developmental abnormalities in animal models, as TMT exposure causes morphological changes in the rodent hippocampus, leading to reduced expression levels of reelin (18), which is an important glycoprotein in the extracellular matrix that is involved with the migration of postmitotic neurons in the cerebral cortex and the synaptic plasticity in the developing brain (19).

Increased levels of reactive oxygen species (ROS), protein carbonyl, and malondialdehyde—biomarkers of protein and lipid peroxidation—were found in the rat hippocampus after TMT exposure (20). Those markers for oxidative stress were followed by behavioral abnormalities. The homeostasis of several antioxidant mechanisms can be altered by TMT in the hippocampus, with a decrease in the expression levels of enzymes, such as catalase, superoxide dismutase (SOD), and glutathione peroxidase (GPx), and an increase in glutathione *S*-transferase (GST), a detoxifying enzyme of which high levels are considered a signal of tissue damage (21, 22). Neuroinflammation is another outcome of TMT intoxication, and several biomarkers, such as activated glial cells and the expression of inflammation-related genes, appear in the brain of TMT-treated rodents. Both astrocytes and microglia release inflammatory cytokines during brain injury process, microglia being mainly responsible for the inflammatory response (23). TMT induces the activation of microglia and astrocytes and leads to the increase of the inflammatory mediators released by them. The accentuated increase of interleukin IL-1β, IL-6, and tumor necrosis factor TNF-α levels in mice hippocampus, as well as iNOS, arginase-1, IL-1β, TNF-α, and IL-6 levels in cultured astrocytes, follows TMT exposure (23). It is also reported that the TMT exposure is capable of impairing the late stages of autophagic flux in primary cultured astrocytes, leading the accumulation of protein aggregates in the cell (24). Genes involved in neuronal differentiation and astrocyte activity, inflammatory response, and apoptosis are overexpressed in the dentate gyrus region of the hippocampus, while such upregulation is not found in the *cornu ammonis* regions (25), adding evidence to the suggestion that TMT intoxication causes specific neuronal damage (26). We can conclude that the hippocampus is an especially TMT-vulnerable structure in the mammalian brain.

Other important studies have been proposed to explain the physiological mechanism of TMT intoxication. Neuropeptide Y and somatostatin are both upregulated in the rat hippocampus in the first 4 days after TMT exposure, correlating to the occurrence of seizures. Treatment with the anticonvulsant phenobarbital blocked the seizures and the upregulation of those hormones (27). Ogita et al. (28) presented some very interesting data that showed an influence of adrenal hormones after TMT exposure. Whereas aldosterone, an important mineralocorticoid hormone, increased TMT cytotoxicity in the mouse hippocampus, the mineralocorticoid receptor antagonist, spironolactone, protected neurons against the TMT effects. The blocking of the glucocorticoid receptor through administration of the antagonist, mifepristone, had effects similar to those caused by aldosterone (28). Similar studies linking both endogenous and exogenous glucocorticoids with the attenuation of TMT brain damage were found in the literature and suggested that the modulation of glucocorticoid receptors interferes with the oxidative stress and cytokine expression related to TMT rodent neurodegeneration models (29–31).

## Tributyltin (TBT) Neurotoxicity

**Figure 1:**
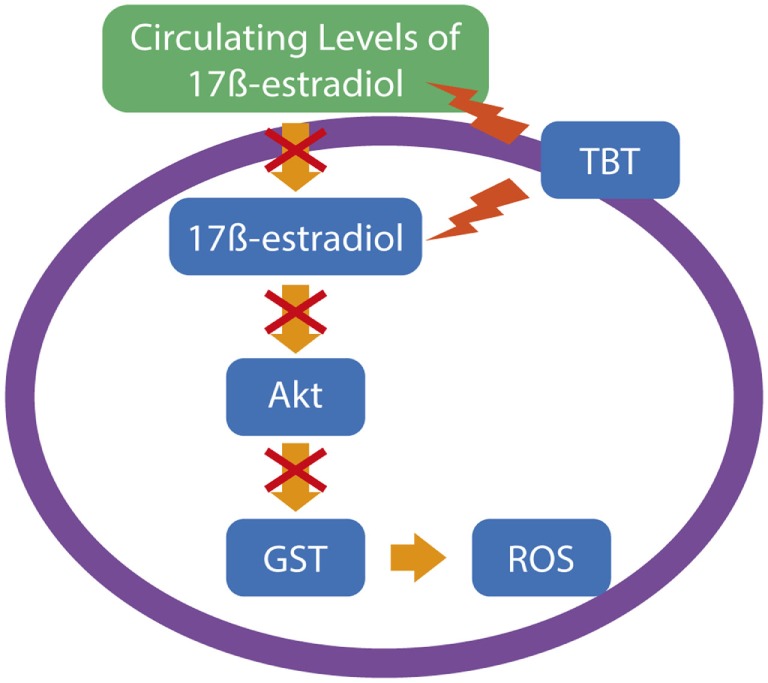
A possible pathway for tributyltin (TBT)-generated oxidative stress in neuronal cells. Considering the data of Ishihara and colleagues (41), supported by data of other groups, TBT leads to neuronal reactive oxygen species (ROS) production by a reduction in the estrogen levels, leading to impairments in the Akt signaling and subsequent downregulation of glutathione *S*-transferase (GST), an important antioxidant mechanism to protect neuronal normal function.

Tributyltin is a highly toxic OT, used initially as a plastic stabilizer and a molluscicide in agriculture, but due to its higher biocide potential, TBT was subsequently extensively used as an antifouling agent in the ships and other marine structures to prevent the adhesion of plankton and other organisms (32). Several studies have reported TBT as a reproductive toxic compound, hepatotoxic, nephrotoxic, and obesogen, and it exerts its toxicity in several groups of organisms (33–36). Neurotoxicity is also described in various experimental organisms and in cultured brain cells after TBT exposure, with oxidative stress and neuroinflammation being a common feature after TBT exposure, which leads to subsequent brain impairments. Mitra et al. observed that TBT-chloride *in vivo* exposure in different concentrations of 10, 20, and 30 mg/kg is capable of disrupting the blood–brain barrier and metal metabolism in the rat brain, followed by protein carbonylation and lipid peroxidation, 4 days after administration. TBT *in vivo* also caused astrocyte activation and overexpression of inflammatory molecules, such as IL-6, Cox-2, and NF-κB. In the same study, TBT *in vitro* led to neurodegeneration and apoptosis by the activation of caspase-3 and -8 (37). Evidence also suggests that TBT induces neuronal damage by suppressing the effects of GST, an important cellular antioxidant mechanism, and subsequently generating ROS (38). The endocrine disruption caused by TBT leads to reduced levels of estrogens by the competitive inhibition of aromatase (39). As the antioxidant role of estrogens in the central nervous system has been reported (40), it is possible to assume that TBT also influences the ROS generation in the brain by suppressing the circulating levels of ovarian estrogen. Ishihara et al. demonstrated that pretreatment with 17β-estradiol is capable to suppress the neuronal injury *via* oxidative stress in cultured hippocampus slices by the activation of the Akt signaling (41). It is interesting to note that, as we cited above, it is suggested that TBT inhibits GST activity, and the decreased activity of Akt in the cardiac tissue and subsequent oxidative stress can be caused by other GST inhibitors (42), suggesting that TBT-induced oxidative stress occurs through GST inhibition *via* Akt deactivation induced by low levels of estrogen in the brain, as summarized in Figure 1.

Rat neurons cultured with astrocytes are less vulnerable to TBT toxicity than neurons cultured purely in primary neuron cultures, indicating that astrocytes play an important role in neuroprotection against TBT toxicity effects (43). Although the hippocampus is one of the main targets of TBT toxicity, considering the deleterious effects following TBT exposure, the striatum seems to be more vulnerable than other brain regions, as ROS generation, protein carbonylation, and lipid peroxidation are more prominent in this region (44, 45). The damage in the hippocampus and the striatum caused by TBT may have cognitive implications, considering that those areas are involved in mammalian memory and learning (46, 47), but further studies in this specific subject are necessary to evaluate this possibility. Based on the presented evidence, we can infer that, like TMT toxicity that targets specific regions of the hippocampal formation, TBT also has differential effects in different brain regions and cell types.

On a cognitive point of view, estrogens are modulators of different types of memory, and their low serum levels due to aromatase inhibition or ovarian failure may be associated with behavioral abnormalities (48). In fact, the brain itself produces estrogens *via* brain aromatase, and this *de novo* synthesis in the hippocampus is fundamental to proper functioning of memory systems (49). In certain animal models, TBT can inhibit not only gonadal aromatase but also brain aromatase, as is observed in the teleost fish. In the Atlantic salmon (*Salmo salar*), a 7-day TBT exposure impaired neurosteroidogenesis, decreasing both the expression of the cytochrome P450 aromatase gene and activity of the expressed aromatase (50). The same gene-suppressive effects of TBT were found in male guppies (*Poecilia reticulata*), where two isoforms of brain aromatase were under-expressed with subsequent alterations in reproductive behavior, after TBT treatment (51). Therefore, the impact of TBT exposure on endocrine systems and its subsequent influence on the nervous system can be diverse.

Evidence suggests that the TBT obesogenic effects are not only due to influences on energy metabolism but also on food intake, as follows. Neuropeptide Y is overexpressed in female rats treated for 54 days with TBT (0.5 µg/kg), and their food intake was also increased (52). TBT exerts its toxicity in other regions of the hypothalamus, as it is shown through the disruption of the rat hypothalamus–pituitary–adrenal axis (53), although there is no current knowledge about the influence of the adrenal hormonal imbalance on brain injury caused by TBT, as reported in TMT intoxication cases. We can conclude that, besides causing obvious anomalies in the reproductive behaviors through alterations in the sexual hormone balance, TBT is also capable of interfere with behavior centers in the hypothalamus.

## Neurotoxicity of Other OTs

Here, we aim to present briefly what is currently known about the neurotoxicity of dibutyltin (DBT), triethyltin (TET), and triphenyltin (TPT), as well as other OTs used in industrial activities (54, 55).

Dibutyltin is a TBT metabolite, and related compounds are used as catalysts in the fabrication of polymers, such as silicone (54). The use of DBT in the polymerization of polyvinyl chloride (PVC) is a focus of special attention, considering that PVC is largely used in water containers and tubes (56). A study with pregnant female rats exposed to DBT (10, 25 ppm, from gestational Day 6 to postnatal Day 21) demonstrated that tin can accumulate in the brain and placenta, is able to cross the placental barrier and is transferred to the offspring (57). TBT is mainly metabolized in the liver, considering the high hepatic concentrations of its metabolites, including DBT, but evidence shows that part of the TBT is also converted into DBT in the brain (58). The brain is highly susceptible to DBT toxicity, and Jenkins et al. showed that DBT causes neuronal death in concentrations 40-fold lower than TMT (59). A sub-chronic exposure to DBT (5, 10, and 20 mg/kg) increased the levels of malondialdehyde, a product from lipid peroxidation, while it decreased the activity of two major antioxidant enzymatic pathways, SOD and GPx, in the rat brain. It was also found to cause DNA damage in the cerebral cortex, probably as a result of oxidative stress. We can assume that, similar to TBT, DBT induces oxidative stress in rat cortical cells (60). The physiological mechanism underlying the DBT-induced oxidative stress is yet to be fully determined. Neurotransmitter systems are influenced by DBT intoxication, as DBT exposure decreased the levels of dopamine and serotonin in the striatum and frontal cortex, respectively, causing impairments in learning and decreased spontaneous locomotion (61). Cholinergic neurotransmission is also affected, as DBT is capable of decreasing the activity of choline acetyltransferase, the uptake of choline into synaptosomes and the myelin content of cholinergic neurons of rodents (62, 63). As the data were obtained through *in vitro* experiments, the behavioral outcomes of the impaired acetylcholine neurotransmission have yet to be evaluated.

Triethyltin is an environmental contaminant that comes from industrial activities, similar to TMT and TBT (55). The main neurotoxic effects of TET, including demyelination of neurons and edema, are already well-described (64–66), although the molecular mechanisms underlying such effects have yet to be discovered. TET-exposed oligodendrocyte cultures presented disruption in the mitochondrial membrane potential, disturbances in the cytoskeleton, and signs of oxidative stress and apoptosis (67), concluding that, besides causing vacuolization of the myelin sheath, TET is also cytotoxic to oligodendrocytes, the glial cells responsible for forming the myelin in the mammalian central nervous system. In addition to oligodendrocytes, astrocytes are also susceptible to TET neurotoxicity (68). The TET influence on cytoskeleton is also described in primary neuron cultures, where it interferes in actin polymerization through imbalances in calcium metabolism, leading to abnormal neurotransmitter release in different neural cell lines (69). Evidence indicates that calcium homeostasis in the brain is sensible to TET exposure, as it is also capable of inducing noradrenaline spontaneous release in rat cultured hippocampal slices by altering the functioning of calcium channels (70).

Triphenyltin was used as a biocide in antifouling marine paints and acts like an endocrine disruptor, impairing the synthesis of estrogens (55). With this in mind, it is safe to assume that TPT exposure is very similar with TBT exposure. TPT is capable of impairing the expression of brain aromatase when administered in certain periods of developments in rats (71). What is remarkable about TPT neurotoxicity is its effects as an excitotoxic compound. In an isolated cell experiment, TPT increased neuronal excitability through alterations in the voltage-dependent Na^+^ current of a hippocampal pyramid cell (72). Another mechanism proposed to explain TPT-induced excitotoxicity is through modifications in glutamatergic transmission by modulation of calcium homeostasis in the pre-synaptic terminal (73). It is not determined yet if calcium modulation in hippocampal neurons by TPT is similar to that observed in TET exposure.

## Concluding Remarks

Although most of the OTs with commercial and environmental relevance share many similarities in their chemical properties, the physiological mechanisms underlying their neurotoxicity are vast and sometimes not fully understood. Each compound has various neuroendocrine and behavioral outcomes in different groups of vertebrates, and a close examination of their biological influence is very important to increase our current knowledge about occupational and environmental health and safety. In summary, OTs are potent neurotoxicants, leading to behavioral impairments due to brain damage in various levels caused, mainly, by oxidative stress and neuroinflammation.

## Author Contributions

IF, LR, and JG: conception of the work. IF: manuscript drafting. LR, JG, and LF-L: critical revision of the work. LR, LF-L, and IF: final version approval.

## Conflict of Interest Statement

The authors declare that the research was conducted in the absence of any commercial or financial relationships that could be construed as a potential conflict of interest.
